# P-647. Symptomatic Presentation and Characteristics of Residents with Test-confirmed Respiratory Infections in a Surveillance Network of US Nursing Homes

**DOI:** 10.1093/ofid/ofaf695.860

**Published:** 2026-01-11

**Authors:** Kevin McConeghy, Lisa Han, H Edward Davidson, Ivis Perez, David Canaday, Stefan Gravenstein

**Affiliations:** COIN-LTSS, Providence Veterans Affairs Medical Center, Providence, Rhode Island; Insight Therapeutics, LLC, Norfolk, Virginia; Insight Therapeutics LLC, Norfolk, Virginia; Insight Theraputics, Norfolk, Virginia; VA Northeast Ohio Healthcare System, Cleveland, OH; Brown University, Providence, RI

## Abstract

**Background:**

Innovations in viral testing may improve nursing home (NH) respiratory pathogen surveillance. Faster and more accurate pathogen detection could help infection control and facilitate case identification and specific antiviral initiation. We describe the findings of pilot placement of a point-of-care (POC) PCR testing device that identifies RSV, Influenza A/B, and SARS-CoV-2 over 2 respiratory seasons.

**Figure:**
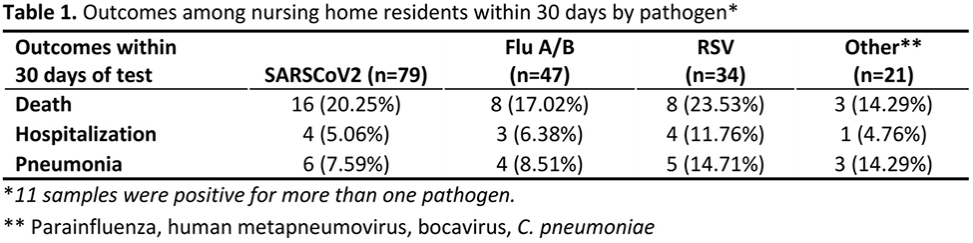

**Figure 1.**
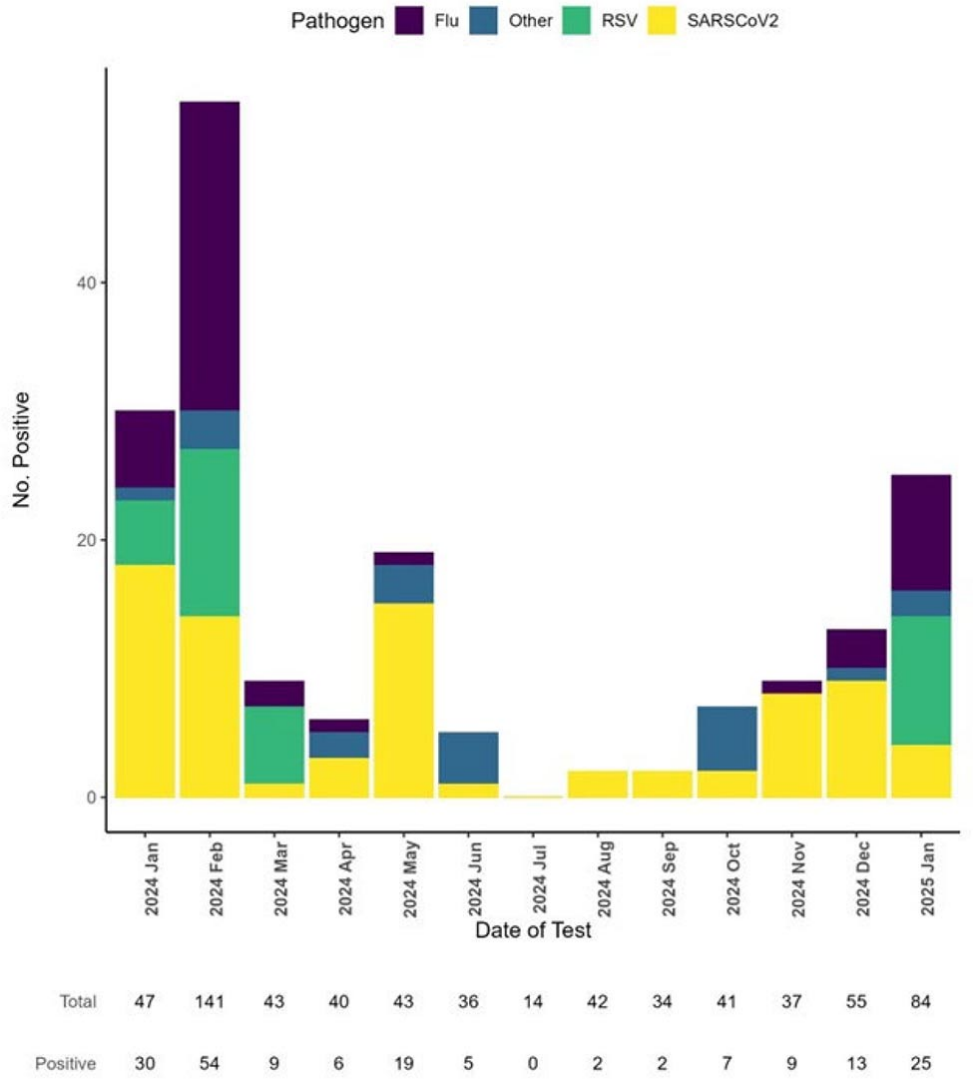
PCR Point-of-Care Testing by Month In A Sample of 23 U.S. Nursing Homes

Graph depicts the number of tests and number positive per month by respiratory pathogen

**Methods:**

We partnered with NH operators to place Cepheid POC PCR devices in NHs to improve respiratory viral testing. This cross-sectional descriptive analysis of infection cases in U.S. NHs spans from Nov 2023—Jan 2025. We supplied 23 NHs with Cepheid® testing devices to use during routine care. We collected residual sample discards for expanded respiratory panel testing with Luminex.® Demographics, chronic conditions, symptoms, and health outcomes of residents were collected through electronic health record (EHR) linkage.

**Results:**

Cepheid and Luminex testing dually confirmed 360 samples for at least one pathogen, 170 of which were from NH long-stay residents, 11/170 reported two pathogens. The other 190 came from short-stay residents, staff, and EHR-unlinked samples. Figure 1 shows distribution of cases over time. The 170 cases included SARS-COV-2 (n=79), Influenza A (n=45)/B(n=2), RSV (n=34), and n=21 other (10 parainfluenza, 9 human metapneumovirus, 1 bocavirus, 1 C. pneumoniae). Mean ages (years) were similar for SARS-CoV-2 (82), Flu (78) and RSV (81). The population was mostly white ( >90%) for all infection types. Flu cases were 66% female versus SARS-CoV-2 (57%) and RSV (41%). Case symptoms differed by fever (36% [Flu] versus 8% [SARS-CoV-2] and 17% [RSV]), and cough/wheezing (71% [RSV] versus 47% [Flu] and 61% [SARS-CoV-2]). Clinical severity for RSV tended to be worse than for SARS-CoV-2 or Flu within 30 days of the Cepheid result (Table 1). Positive tests for SARS-CoV-2 were prevalent throughout 2024, while Flu and RSV peaked in January/February.

**Conclusion:**

This surveillance network highlights the unique presentations and health outcomes of infectious pathogens in NH residents. Rapid molecular testing may play a key role in identification, prevention, and treatment of respiratory infections which cause significant NH morbidity.

**Disclosures:**

Kevin McConeghy, Pharm.D., GlaxoSmithKline: Investigator-Initiated Study|Moderna: Grant/Research Support Lisa Han, MPH, GSK: Grant/Research Support|Moderna: Grant/Research Support|Sumitomo: Grant/Research Support H Edward Davidson, PharmD, GSK: Grant/Research Support|Moderna: Grant/Research Support|Sumitomo: Grant/Research Support Ivis Perez, MPH, LPN, GSK: Grant/Research Support|Moderna: Grant/Research Support|Sumitomo: Grant/Research Support David Canaday, MD, Moderna: Grant/Research Support|Pfizer: Grant/Research Support|Seqirus: Advisor/Consultant|Seqirus: Grant/Research Support|Seqirus: Honoraria Stefan Gravenstein, MD, MPH, GSK: Advisor/Consultant|GSK: Grant/Research Support|GSK: Honoraria|Moderna: Grant/Research Support|Novavax: Advisor/Consultant|Novavax: Honoraria|Pfizer: Advisor/Consultant|Pfizer: Grant/Research Support|Pfizer: Honoraria|Sanofi: Advisor/Consultant|Sanofi: Grant/Research Support|Sanofi: Honoraria|Seqirus: Grant/Research Support

